# Hybrid three-dimensional full-view multi-wavelength photoacoustic and ultrasound breast tomography^☆^

**DOI:** 10.1016/j.pacs.2026.100847

**Published:** 2026-06-13

**Authors:** M. Dantuma, F. Lucka, S.C. Kruitwagen, L. Alink, R.P. Pompe van Meerdervoort, M. Nanninga, T.J.P.M. Op ’t Root, B. De Santi, E. Bosman, J. Budisky, G. Bordovsky, E. Coffy, M. Wilm, T. Kasponas, S.H. Aarnink, L.F. de Geus-Oei, F. Brochin, T. Martinez, A. Michailovas, W. Muller Kobold, J. Jaros, J. Veltman, B. Cox, S. Manohar

**Affiliations:** a Multi-Modality Medical Imaging group, TechMed Centre, University of Twente, Enschede, the Netherlands; b Computational Imaging, Centrum Wiskunde & Informatica, Amsterdam, the Netherlands; c Department of Computer Science, University College London, London, United Kingdom; d Medisch Spectrum Twente Hospital, Enschede, the Netherlands; e Department of Medical Physics and Biomedical Engineering, University College London, London, United Kingdom; f P.A. Imaging R&D B.V., Enschede, the Netherlands; g Department of Computer Systems, Faculty of Information Technology, Brno University of Technology, Brno, Czech Republic; h Imasonic SAS, Voray-sur-l’Orgnon, France; i Ekspla UAB, Vilnius, Lithuania; j Biomedical Photonic Imaging group, University of Twente, Enschede, the Netherlands; k Department of Radiology, Leiden University Medical Center, Leiden, the Netherlands; l Center for Physical Science and Technology, Vilnius, Lithuania; m Department of Radiology, Ziekenhuisgroep Twente, Hengelo, the Netherlands

**Keywords:** Photoacoustic tomography, Optoacoustic tomography, Ultrasound tomography, Breast imaging, Hybrid imaging, Ultrasound transducers, Speed of sound, Full waveform inversion

## Abstract

Photoacoustic tomography is a contrast agent-free imaging technique capable of visualizing blood vessels and tumor-associated vascularization in breast tissue. While sophisticated breast imaging systems have been recently developed, there is yet much to be gained in imaging depth, image quality and tissue characterization capability before clinical translation is possible. In response, we have developed a hybrid photoacoustic and ultrasound tomographic system (PAM3). The photoacoustic component has for the first time, in a full-view hemispherical breast system, three-dimensional multi-wavelength imaging capability and implements substantial technical advancements in critical hardware and software sub-systems. The ultrasound component enables three-dimensional ultrasound (computed) tomography from both reflected and transmitted signals from which we currently extract an image of the sound speed. For the first time, *in vivo* sound speed images were reconstructed using a fully 3D full-waveform inversion (FWI) algorithm, which demonstrated excellent quantitative as well as spatial accuracy. The sound speed image of the breast was then incorporated in photoacoustic reconstruction to correct for acoustic inhomogeneities, enabling accurate target recovery. The results demonstrate identification of blood vessels to depths of up to 48 mm, with a more uniform field of view than hitherto, and an isotropic spatial resolution comparable to the in-plane resolution of clinical breast Magnetic Resonance Imaging. The *in vivo* performance achieved, and the complementary diagnostic value of interrogating angiogenesis-driven optical contrast as well as tumor mass sound speed contrast, gives confidence in the system’s clinical potential.

## 1 Introduction

Photoacoustic (PA) tomography, is a non-invasive imaging modality that relies predominantly on differential optical absorption of tissue constituents [1], [2] for physical contrast. Unlike in purely optical techniques where light that survives the absorption is measured for the signal, in PA a surrogate of the actual absorbed nanosecond pulse optical energy is detected, namely acoustic waves [3]. These waves in the ultrasound (US) frequency regime are launched by transient thermoelastic expansion when the absorbed optical energy is converted into heat by non-radiative deexcitation processes. Since the propagating US wavefronts in soft tissue experience relatively low scattering compared to light, their faithful detection at the tissue surface enables accurate localization of the often deeply located US sources. The method is thus attractive since it combines the ability to interrogate intrinsic optical spectroscopic contrast of tissue components, with the ability to localize this contrast deep inside soft tissues with high resolution [4], [5], [6], [7], [8].

PA tomography is interesting for investigating the female breast due to the organ’s relatively low acoustic scattering and absorption, as well as its accessibility for light excitation and US detection from all directions when pendant. Importantly the breast is the site of the highest incidence of female cancers globally, with 2.3 million new cases and 685,000 deaths in 2020 [9], while established breast imaging methods suffer from drawbacks and limitations. X-ray mammography uses ionizing energy, applies often painful breast compression, and shows poor performance in visualizing cancer in breasts rich in fibroglandular tissue [10], [11], [12]. US imaging has poor discrimination between malignancies and benign abnormalities in the evaluation of solid masses [11], [13]. Magnetic Resonance Imaging (MRI) shows excellent sensitivity by mapping contrast agent kinetics in tumor vasculature, but there is considerable overlap in this behavior between malignancies and benign abnormalities [11], [14]. The method also requires contrast agents, is expensive, and not universally available.

PA tomography uses non-ionizing radiation, has low burden to the patient and has been demonstrated to be able to image blood vessels associated with breast cancer with high resolution, without the use of contrast agents [15], [16], [17]. Various PA breast imaging configurations have been developed, determined by the US detection aperture, and can be roughly divided into four geometries [15]: linear [18], planar [19], [20], curved/circular [21], [22] and hemispherical [23], [24]. Of these, the last mentioned geometry is most appealing because the US detectors can capture the PA emissions over a solid angle of 2π steradians, which gives sufficient data to reconstruct any object within the hemisphere without limited view artifacts [25], [26], [27].

The latest and most sophisticated dedicated hemispherical geometry PA breast imager was presented in 2021 by Lin et al. [5]. The imager, using a single wavelength of 1064 nm and US detection with 1024 elements, revealed detailed vascular anatomy in the breast with an isotropic spatial resolution of 0.37–0.39 mm, to depths with a maximum of 40 mm and with an imaging time of 10 s per breast [5]. The system represents one of the most advanced dedicated breast PA computed tomography platforms reported to date but still employs non-uniform illumination at a single wavelength, which impacts on the accuracy of imaging and the ability to extract quantitative spectral information. Further, this imaging system and its image reconstruction, as all others before it [28], [29], [30], [31], treat the breast as being acoustically homogeneous. A more generalized tomographic imaging platform was presented by Zhang et al. [24], where in addition to PA detection, reflection-mode UST was implemented using transmission from a single US transducer. In breast imaging experiments, this system showed an imaging depth of approximately 32 mm in the one subject studied. However, the same limitations as noted above remain, including single-wavelength operation and the assumption of acoustic homogeneity.

While PACT offers unique sensitivity to optical absorption contrast and thus making it possible to image vascular architecture and physiology, several challenges remain that limit its clinical translation. In particular, quantitative interpretation is complicated by unknown optical fluence distributions, and image quality can be significantly degraded by acoustic heterogeneities in tissue. These limitations motivate the development of approaches that explicitly account for acoustic properties, such as the sound-speed imaging and correction framework presented in this work.

In this paper, we present a hybrid breast imaging system (PAM3) that has multi-wavelength PA imaging capability in hemispherical geometry, as well as for the first time the capability to measure a fully three-dimensional (3D) sound speed image of the breast through hybrid ultrasound tomography (UST). Sound-speed images are reconstructed using a full-waveform inversion (FWI) approach. In contrast to time-of-flight–based (TOF) approaches, which only make use of the transmitted US signals and rely on ray-based wave-propagation approximations resulting in limited spatial resolution, FWI uses a more accurate and complete model of wave propagation and makes use of all recorded US signals, enabling more accurate and higher-resolution sound-speed reconstruction in the complex breast [32]. The reconstructed sound speed image is used to correct the wavefront aberrations experienced by the PA waves as they traverse the breast. This enables highly accurate recovery of the PA sources and contributes to deeper imaging performance. A standard image acquisition protocol uses 5 wavelengths and takes 5 min. The PA tomography mode is a major technological improvement in itself due to advancements in sub-systems such as the laser, light delivery system, ultrasound transducers, data acquisition (DAQ) and control systems electronics, and image reconstruction algorithms. We present details of the PAM3 system, a comprehensive characterization of its capabilities on a specially-developed suite of test-objects and sophisticated phantoms, and demonstrate its performance on healthy breasts.

## 2 Methods

### 2.1 Photoacoustic Tomography

The PA images are estimated for each excitation wavelength from the measured PA time traces using an iterative, model-based image reconstruction method which is expected to lead to quantitatively accurate solutions [33]. It relies on a numerical wave propagation simulation that can use three different SOS models, reflecting an increasing degree of accuracy: Using the SOS of water at 25°C (which corresponds to the coupling medium in the bowl) everywhere in the volume will be called “1-SOS model” and does not account for SOS heterogeneities [38]. Using a binary mask of the breast cup to assign a different SOS value inside the breast will be called “2-SOS model” [38]. The value inside the breast can be manually or automatically tuned to obtain sharp blood vessels. Using the SOS image reconstructed from the US measurements (see Sec. “Ultrasound Tomography” below) to compensate for SOS heterogeneities will be called “full-SOS model”. The implementation of the numerical wave propagation is based on the k-Wave toolbox [34], but uses a tailor-made CUDA implementation that also includes part of the US transducer modeling for memory efficiency. The US sensing performance of the transducers is modeled to account for their directional response and the empirically measured temporal impulse responses and relative sensitivities of all the elements. See Sec. “US transducer characterization”, for the corresponding calibration measurements. All PA images in this manuscript were reconstructed on an isotropic computational grid of 0.4 mm. Single forward and adjoint [35] wave simulations on this grid take 3 min and 9 min respectively, to run on an NVIDIA A100 GPU and supports frequencies up to 1.87 MHz, which means that all the relevant detector bandwidth is covered. The total run time of reconstructing one PA image takes around 2 h 30 min. Further technical and implementation details will be described in a forthcoming publication.

### 2.2 Ultrasound Tomography

The SOS images are estimated from the US time traces using the full-waveform inversion (FWI) scheme described in Ref. 36: FWI is a non-linear, iterative, model-based image reconstruction method that optimizes the SOS image to match the recorded data using a numerical wave propagation scheme. The latter is the same wave propagation model used in the PA tomography. Smoothed Total Variation (TV) regularization and bound constraints ranging from 1400 m/s to 1600 m/s are used to regularize the solution. A stochastic gradient descent scheme with inertia and progressive iterate averaging is used as the optimization scheme and the squared slowness transform is used as preconditioning. In each iteration, four source encoding gradient estimates are averaged that combine the US data from four different rotational positions. While only the SOS is directly optimized for with gradient updates, the corresponding acoustic density (in kg/m^3^) of the medium is fixed to 0.76 × SOS (in m/s)based on the empirical correlation between density and SOS in soft tissues. The FWI is embedded in a coarse-to-fine multigrid scheme running on spatial grids of resolution 2 mm, 1.5 mm and 1.0 mm successively. On each grid, 128 iterations are run, which takes 1 h/2 h/7 h for the three grids respectively, on a server with 4 NVIDIA GeForce RTX 2080 Ti GPUs (total FWI run-time 10 h 6 min) using non-optimized Matlab code. For the experimental phantom data described in Sec. “Validation of SOS imaging“, we also extended the multigrid scheme by doing 64 iterations on 0.8 mm and 0.6 mm resolution, which takes 12 h and 46 h on a server with 2 NVIDIA RTX A6000 GPUs. Further technical, implementation and calibration details will be described in forthcoming publication. To initialize the FWI on the coarsest grid, an SOS image reconstructed by a time-of-flight (TOF) method is used that is described in the supplementary material.

### 2.3 US transducer characterization

The characterization measurements were performed in an external set-up on all 512 US elements before the elements were installed in the imaging bowl. Each US element was connected to a 40 dB low-noise amplifier. The impedance of amplifier and the cable lengths used in these characterization measurements were similar as in the PAM3 imager.

Noise measurements were performed with a spectrum analyzer (Agilent 8594E) in the 0.2–2 MHz frequency range. The US element was placed in a Faraday cage to minimize environmental noise. The US element electrical impedance was measured with a network analyzer (Agilent 4294) in the same frequency range and it was checked that the noise measurement results fit well with a theoretical calculation based on the transducer impedance (thermal noise) and the amplifier noise.

The sensitivity of the US elements was measured with a substitution method. The transmitter was a large bandwidth transducer. The central frequency and bandwidth of the reference transducer are: *f*_c_ = 1 MHz, *f*_l_ = 0.592 MHz and *f*_u_ = 1.50 MHz −6 dB for a bandwidth of nearly 1 MHz. (*f*_c_ is center frequency, *f*_l_ is lower frequency and *f*_u_ is upper frequency)

The reference transducer was excited with a chirp signal (0.1–2 MHz bandwidth) with a generator (Keysight 33600 A, USA). The incident pressure produced by the transmitter was measured with a PVDF hydrophone (Onda HGL 0400). This hydrophone was then replaced by the US element connected to the 40 dB amplifier and the generated voltage (*V*_out_) on the US element was recorded. The receive transfer function (RTF) (V/Pa) was calculated from the incident pressure *P*_*in*_ and the measured voltage (*V*_out_) using RTF = 20 log (*V*_out_*/P*_in_).

The frequency responses of all transducers were measured after installing the detectors in the system. In this case, all elements were characterized simultaneously by means of a PA measurement on a specially designed black phenolic ball [37] at 755 nm with 121 bowl positions and 16 measurements per position (averages). All the time-traces measured with one detector (121 ×16 signals) were shifted in time to have all peaks overlapping. These shifted time traces were averaged, and the frequency response was calculated for each detector by Fourier transforming this averaged signal.

### 2.4 Light distribution on the cup surface

A set of PA measurements was performed on black variants of the eight breast-supporting cups [37], [38] to visualize the light distribution on the surface of each. The black cups were produced by vacuum forming a 1 + /- 0.1 mm thick black PVC-U sheet over the same moulds that are normally used to produce the transparent breast-supporting cups [38]. The cups were taped to the same metal rings that are normally taped to the disposable transparent cups. With this ring, the cups were installed one-by-one in the imaging aperture and a PA measurement at 755 nm using 201 bowl positions and 10 PA averages was performed on each of the cups.

### 2.5 Spatial resolution measurements

A test-object was developed to measure the PA spatial resolution over the entire imaging volume in the three imaging dimensions. The object contains 277 sub-resolution India ink coated stainless steel microspheres (Cospheric LLC, United States) that have a 310–360 µm diameter which are distributed throughout a cup 8 vol and held in a polyvinyl plastisol (PVCP) matrix [37]. The PVCP (LureFlex firm, LureFactors, UK) has a 1403 ± 3 m/s sound speed at room temperature and at 1 MHz and is optically and acoustically semi-transparent. A highly sampled PA measurement was performed on this object, consisting of 401 bowl rotations and 30 averages. Reconstructions were made with the full dataset but also with down sampled sets of the data to investigate the effect of the number of bowl positions, averages and reconstruction iterations on the spatial resolution.

### 2.6 Validation of SOS imaging

A sound speed test object was developed consisting of two main tissue mimicking regions, namely the adipose and fibroglandular region. Cylindrical rods (4, 6, 8, 10 mm diameter) filled with water were embedded in the glandular region to assess spatial and contrast resolution. The acoustic properties of the materials used in the sound-speed test object were characterized at 24.9 ± 0.2 °C using transmission measurements based on a modified insertion method [39]. More details are provided in Supplementary Materials. The resulting sound-speed values, calculated as described in Ref. 39, were used as reference values for validation of the UST–based sound-speed imaging in the PAM3 system. Water in the imager was measured to have a temperature of 24.9 ± 0.1 °C. Imaging was performed with all 512 transducers emitting at each of the 101 bowl positions, which resulted in the maximum amount of US shots within the 10 min acquisition time limit of the DAQ. The SOS image was reconstructed using FWI as described above.

### 2.7 Volunteer recruitment and measurements

The Medical Research Ethics Committees United (MEC-U), acknowledged by the Dutch Central Committee for research involving human subjects, reviewed and approved the study. An independent party (DARE!! Measurements, The Netherlands) performed product safety tests on the PAM3 system, which the local hospital used to approve the release of the imager for measurements on human subjects. The study was registered in the Netherlands National Trial Register under the identification number NL7992.

In total, eight healthy volunteers, aged between 23 and 64 years, were recruited for the study. The most important exclusion criteria included pregnancy or breastfeeding, a history of breast disease, and the presence of a tattoo or piercing in the breast area. Each volunteer was informed about the study and informed consent was obtained in all cases. For each volunteer, a short questionnaire was used to classify the skin tone on the Fitzpatrick scale [40]. The skin types and the questionnaire are included in Supplementary materials (Tables A3 and A4)

Multiple measurements were performed on each volunteer, within a maximum measurement time of 20 min per breast. A standard PA-US measurement protocol consisting of 101 bowl positions takes 5 min for 5 wavelengths with 4 averages, but since measurement sequences were not the same for all volunteers, individual measurement times are not the same. The number and choice of wavelengths, number of bowl rotation steps and number of PA averages are examples of settings in the sequence that were varied in the study to investigate how these parameters influence the results, from which the settings for optimal imaging quality were extracted.

For every measurement, the appropriate cup size was selected, and the cup was installed in the imaging aperture. The breast to be imaged was disinfected and the volunteer positioned prone on the bed with her breast in the cup. The volunteer wore laser goggles as protection against possible laser light escaping from the bowl. The arm contralateral to the breast being imaged was placed above the head, and the contralateral leg was bent outwards to incline the torso. This specific way of positioning increased the volume of breast tissue available in the imaging bowl. Pillows for the head, the hips and the ankles were available and were used in case the volunteer experienced discomfort. Once the volunteer was comfortable, the operator at the control PC made sure that the water level in the bowl was topped off and then started the measurement. The described protocol was repeated for the contralateral breast. After the measurements, both breasts were visually inspected by the operator for birthmarks, scars and other skin markings, and their locations noted.

### 2.8 Image analysis and visualization

#### 2.8.1 Spatial resolution calculation

Additional information can be found in the document ‘Supplementary Materials’.

#### 2.8.2 Visualization of in-vivo PA images

No motion correction was employed since a first investigation revealed no discernable breathing artefacts in the PA images. For image visualization, the 3D reconstructed volumes were first Frangi-filtered to enhance the contrast of the vessels [41], [42]. Then adaptive intensity modulation (AIM) [42] was applied to homogenize the voxel intensities by suppressing the strong superficial voxels and enhancing the deep low-intensity structures. AIM was implemented by dividing the Frangi-filtered image by a standard deviation map that was calculated by sliding a 10x10x10 voxel window over the image in all dimensions with a 3-voxel step size [43]. AIM was applied only for visualization purposes and not used in any of the analyses involving spectral or quantitative interpretation. The final image was a weighted summation of the original image and the intensity modulated image [5], [43]. Weighting factors that gave the visually most compelling images were used and yielded 0.6 and 0.4 respectively.

For depth-encoding the images, we made use of the well-defined breast position and cup contour [38]. This allowed the shortest distance to the breast surface to be calculated for each voxel within the cup volume. A 2D colormap was prepared with color hues on one axis representing the depth from the skin and the color value on the second axis representing the PA intensity.

#### 2.8.3 Visualization of UST images

For image visualization, the 3D reconstructed SOS volumes are sliced (typically x = 0, y = 0 and/or selected z slices) and displayed using a gray colorscale, which ranges from 1400 to 1600 m/s for the in-vivo data.

## 3 Additional system information

The document ‘Supplementary materials’ carries more information about the system including the laser, light delivery, system control and data storage.

## 4 Results

### 4.1 System description

**Fig. 1:**
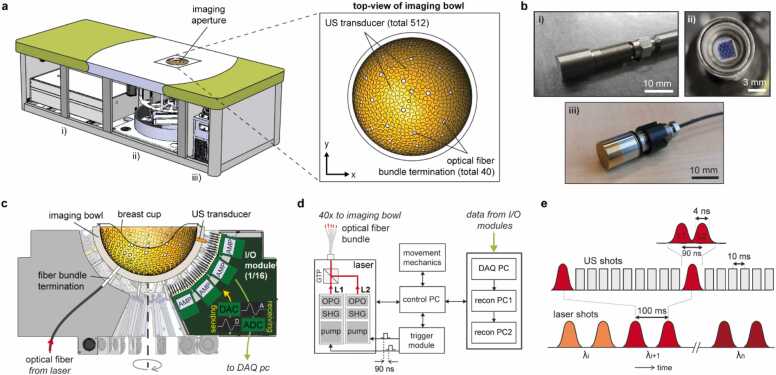
Overview of the PAM3 breast imaging system. The subject lies in a prone position with one breast through the imaging aperture into the temperature-stabilized water-filled imaging bowl. The bowl has two sets of bowl-inserts namely ultrasound (US) transducers and optical fiber bundle terminations. **a,** Illustration of the system in perspective view with one of the side panels removed to reveal three hardware compartments containing i) the laser, ii) the rotating turret and iii) temperature control unit. Inset is the top-view of the imaging bowl showing the arrangement of the bowl-inserts. **b,** Photographs of i) a fiber bundle termination, ii) the output microlens array termination of an optical fiber bundle, and iii) an US transducer. **c,** Cross-sectional view of the core of the system, showing the imaging bowl, and I/O modules arranged around it. There are in total 16 I/O modules each comprising 32-channel Analog-Digital converters (ADC) and Digital-Analog converters (DAC) to service the 512 US transducers. **d,** Block-scheme showing various modules, their interconnections and signal transfers. **e**, timing diagram of laser and US firing for a measurement sequence consisting of n wavelengths and two PA averages. AMP = pre-amplifier, recon = reconstruction, DAQ = data acquisition, L1 = laser head one, L2 = laser head 2, OPO = optical parametric oscillator, SHG = single harmonic generator, GTP = Glan-Taylor polarizer.

The PAM3 system is built into a custom-designed frame with bed-top (2.5 m × 1 m size) for the woman to lie on prone, with one breast through the imaging aperture which leads into a water-filled imaging bowl beneath. Fig. 1a is an illustration of the system with a side panel removed to show the hardware groups under the bed-top containing respectively: i) the laser system, ii) the rotating core of the system, and iii) the water-temperature-control system. The top-view of the imaging bowl, as seen through the aperture, is shown in the inset of Fig. 1a. Supplementary Fig. A1 shows photographs of the system and the imaging bowl. The hemispherical bowl has a 26 cm inner diameter and has holes arranged in a spiral fashion to accommodate the following water-tight inserts: 40 optical fiber bundle terminations to provide light for PA excitation, 512 single-element transducers for the detection and emission of ultrasound, and two platinum resistance thermometers to measure water temperature. Each fiber bundle termination (Fig. 1b) comprises a fused silica micro lens array to shape the beam into a top-hat profile and ensure that the beam diverges rapidly to achieve a good coverage of the breast. The US transducers (Fig. 1b) (Imasonic, Voray-sur-l’Ognon, France) have a 1 MHz center frequency, and each comprises a 3 mm diameter piezocomposite active element which can be used both in detection and emission modes.

Fig. 1c shows a cross-section of the rotating core of the PAM3 system, and Fig. 1d is a block-diagram showing various modules with their coupling and data transfers. An optically and acoustically transparent polyvinyl chloride (PVC) cup [38] is used to centralize and stabilize the breast in the bowl, and mitigate motion of the breast during a measurement. The breast-supporting PVC cups have an approximate thickness of 180 *µ*m, with holes to ensure that the breast is in contact with water for good acoustic coupling with the US transducers.

The tunable wavelength PA excitation source (Ekspla UAB, Vilnius, Lithuania) comprised two identical units each consisting of a Q-switched Nd:YAG pump laser, a DKDP crystal second harmonic generator (SHG), and a BBO crystal optical parametric oscillator (OPO) producing pulses of 5 ns duration. In normal operating mode, the pump lasers are triggered with a 90 ns delay between them, to avoid overlap of the two pulses in time to stay under the damage threshold of the fiber bundle (see Fig. 1e). This delay corresponds to an acoustic travel of approximately 0.14 mm, which is below the in vivo resolution (0.75 mm), and therefore does not produce duplicate structures which can be resolved. The two OPO outputs are combined using a Glan-Taylor prism (GTP) into a single beam coupled to the fiber bundle. .The fiber bundle possessed a single aperture at the bundle entrance, and the output was arranged as 40 smaller aperture endings with nearly equal output energy distribution. ·

The US transducer cables lead to input-output (I/O) electronic modules. During a measurement, the bowl together with the inserts, their connections and couplings, and the I/O modules, rotates step-wise over 360 ° to acquire multiple-views around the breast. The spiral arrangement of the US transducers ensures that their positions remain unique during a full rotation. Each I/O electronics module (PA Imaging R&D B.V., Enschede, The Netherlands) services 32 US transducer elements and comprises a Data Acquisition (DAQ) part and an US Actuation part. The system control software allows individual measurement sequences to be programmed (number of bowl rotational steps, number of wavelengths, number of averages, number of US shots). The timing diagram of an example measurement sequence consisting of 5 wavelengths, two PA shots and 9 US shots is illustrated in Fig. 1e. This sequence is repeated at each bowl rotational position.

### 4.2 Light delivery, ultrasound detection and breast supporting cups

**Fig. 2:**
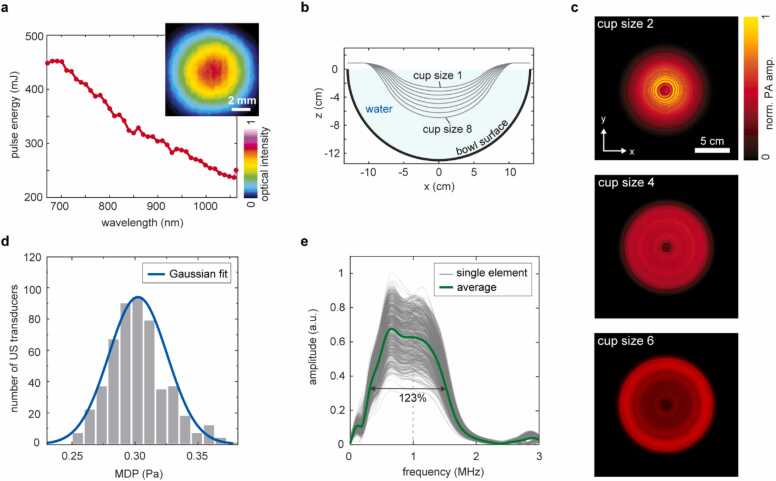
Characteristics of the custom-developed laser unit, light delivery approach, US transducer elements, and breast-supporting cups. **a,** The laser pulse energy as a function of wavelength. The inset shows the beam profile at 800 nm at a distance of 23 mm in air from one of the 40 fiber bundle terminations. **b,** Radially symmetric contours through the centers of the eight breast-supporting cups. **c,** Maximum intensity projections (MIP) of PA reconstructions of a selection of 3 black breast-supporting cups in the imaging bowl. The uniform reconstructed intensity shows that the cups and thus the breast surface receives a homogeneous radiant exposure. **d,** Histogram of the minimum detectable pressure (MDP) distribution of the 512 US transducers. A Gaussian fit of the data gives a mean MDP at 0.3 Pa. **e,** The measured frequency responses of the 512 US transducers showing an average fractional frequency bandwidth of 123% around 1 MHz.

The output energy of the PA excitation system varies from 450 mJ at 680 nm to 230 mJ at 1060 nm (Fig. 2a). This combined energy is roughly 3 times higher, and each laser-OPO’s energy 1.5 times higher, than the reported energy from lasers in the same class. The inset shows the output intensity profile at 800 nm of one of the 40 optical fiber bundles measured at 23 mm distance in air with a beam profiler (DataRay Inc, Redding, USA). In the imaging bowl, the beam radiates into the water with a full opening angle of 32 °. The arrangement of the fiber bundle endings aims for a homogeneous illumination profile on the breast surface (Supplementary Fig. A2, Table A1) while keeping the radiant exposure on the breast below the Maximum Permissible Exposure (MPE) for skin at 10 Hz. When an appropriately-sized breast-supporting cup is selected from the eight sizes available (Fig. 2b), for a measurement on a human subject, the breast fills the cup and takes its shape. The light distribution on the breast surface can be therefore visualized with PA measurements on light absorbing breast-supporting cups made from 1 mm thick black unplasticizied PVC. The appearance of the PA reconstructions (Fig. 2c) as maximum intensity projections (MIPs) of measurements on different cup sizes show the radiant exposure on the cups and thus the breast. The US transducers were measured to have an average minimal detectable pressure (MDP) of 0.30 ± 0.02 Pa, (center frequency 1 MHz and 123% fractional frequency bandwidth), the lowest reported in the literature [44], [45] (Fig. 2d). The center frequency lies around 1 MHz with a 123% fractional frequency bandwidth (Fig. 2e).

### 4.3 Test objects and phantoms imaging

**Fig. 3:**
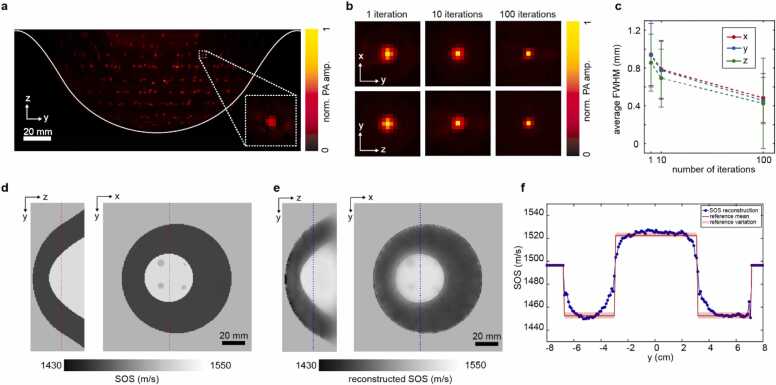
Photoacoustic (PA) and speed of sound (SOS) imaging performance on test objects and phantoms. **a,** A maximum intensity projection (MIP) from the PA measurement of the PSF test object. The inset shows a zoom-in on one of the beads showing the isotropic nature of the PSF. **b,** MIPs of the average PSF of all the reconstructed beads in the test-object for 1, 10 and 100 reconstruction iterations in the xy and yz plane. This shows that increasing the number of reconstruction iterations narrows the PSF. **c,** The spread and mean of Gaussians fitted to all PSFs in the test object for 1, 10 and 100 iterations. **d,** Reference sound speed distribution in the SOS test object in *xy* and *xz* planes. The SOS values were measured on reference objects using transmission measurements in an independent characterization set-up. **e,** Slices of the reconstructed SOS map at the same locations as in **d**. **f,** The sound speed profiles along the dashed lines in **c** and **d**.

The imaging performance of the PAM3 system was assessed and optimized through a series of measurements on a specially developed suite of test objects and phantoms [37], [39], [46]. The 3D spatial resolution was assessed by imaging a test object consisting of multiple sub-resolution (± 300 *µ*m diameter) PA targets [24] embedded in polyvinyl chloride plastisol (PVCP) in a size 8 breast cup (Supplementary Fig. A3). Fig. 3a shows the MIP along the *x*-axis of the PA reconstruction of the test object as described in the previous sections. It uses a 2-SOS model utilizing the known object shape and homogenous SOS. The dashed box marks a selected point target of which a zoomed-in view is shown. Increasing the number of reconstruction iterations appears to narrow the PSFs of the beads. This effect is visualized in Fig. 3b, where MIPs of the mean PSF of all the point targets inside the test-object for 1, 10 and 100 PA reconstruction iterations are shown in the *xy* and *yz* plane. For 10 reconstruction iterations and 101 bowl rotational steps, FWHMs of 786 *µ*m in *x*, 775 *µ*m in *y* and 693 *µ*m in *z* directions were estimated. The spatial resolution can be improved to 426 *µ*m in *z* when 100 reconstruction iterations are used in combination with 101 or more bowl rotational steps (Fig. 3c).

The SOS imaging performance of the system was evaluated with measurements on a well-characterized sound speed test object. Fig. 3d shows two slices of the reference sound speed image of the test object in the imaging bowl, in which the outer adipose and inner glandular layer and the four rod-shaped channels are clearly visible. The construction of this reference image is described in detail in the Supplementary Material. The corresponding slices of the SOS images (Fig. 3e), reconstructed from the UST mode measurements using the 3D FWI method [36], reproduce the composite structure of the test object with minor blurring over the compartment boundaries. Three of the water-filled channels within the object (6, 8, and 10 mm in diameter) are distinctly resolved, whereas the 4 mm channel shows reduced visibility due to its size and vicinity to the adipose-glandular boundary, reflecting the resolution limit of the FWI approach on the 1 mm spatial grid. Fig. 3f shows the SOS profiles along the dashed lines in Figs. 3d and 3e. While the quantitative SOS values inside the compartments are closely matching the range of the reference measurements, one can see spatial extent of the blurring across compartment boundaries. Overall, the mean and median absolute deviation of the SOS reconstruction of this profile measured inside the phantom compared to reference is 5.1 m/s and 2.5 m/s respectively. This corresponds to 2.6% and 1.2% respectively of the sound speed contrast range (1400–1600 m/s). In the Supplementary Material, we show that extending the FWI to a 0.6 mm spatial grid further improves sharpness, contrast, and quantitative accuracy (Figure A6).

### 4.4 In vivo imaging

We imaged both breasts each of eight healthy female volunteers; four exemplary breast images of four individuals are included, of which we discuss two in detail. PA and SOS images of all volunteers can be found in Supplementary Fig. A4. Detailed information about the parameter setting can be found in Supplementary Table A2.

Volunteer 1 was a 64-year-old woman (brassiere size 85 A), who had best fit to breast cup 3. Volunteer 2 was 51 years old (brassiere size 90D) and breast cup 6; volunteer 3 was 55 years old (brassiere size 90D) and breast cup 8; volunteer 4 was 58 years old (brassiere size 80 C) with breast cup 5. All four volunteers had skin tones matching category 2 out of 5 on the Fitzpatrick scale [40] (Supplementary Tables A3 and A4).

**Fig. 4:**
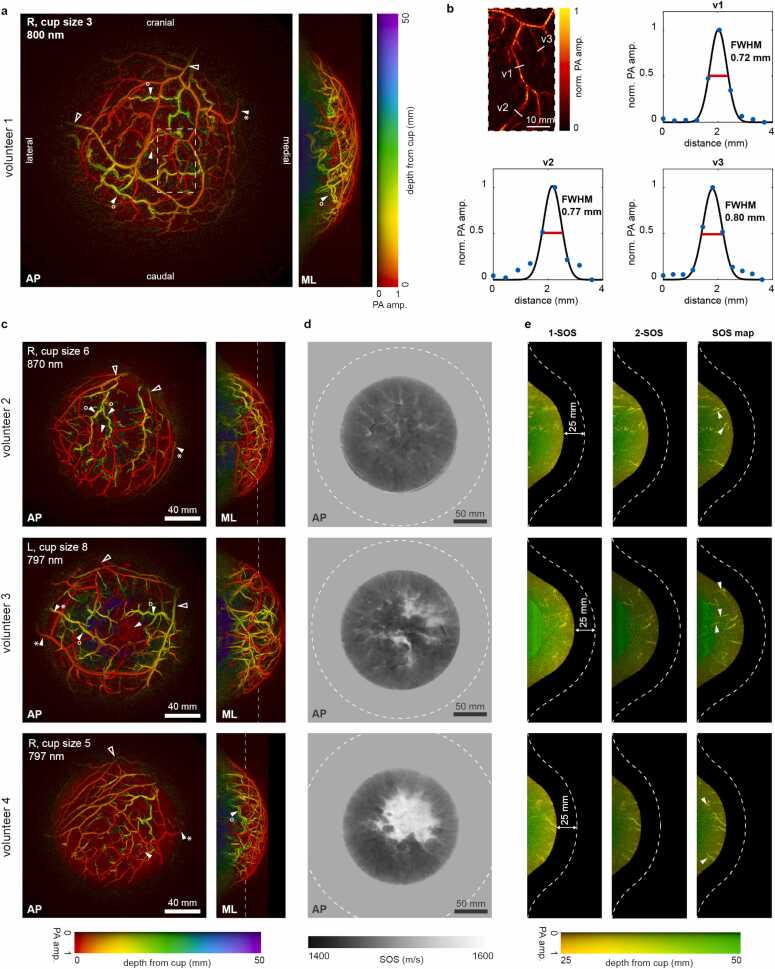
In vivo PA and SOS breast images made with the PAM3 system **a,** full-SOS compensated AP, ML, and CC PA MIP images of the right breast of healthy volunteer 1, demonstrating the system’s capability for full breast imaging. **b,** A zoomed-in view of the superficial vessels (0–0.35 cm deep) in the ROI that is indicated by the white dashed box in a. Cross-sectional profiles of three vessels (v1, v2 and v3) are plotted and fitted with Gaussians to measure the vessel diameters. **c,** full-SOS compensated AP and ML PA MIPs of volunteers 2, 3 and 4 to further demonstrate the performance of the system. Locations of the nipple (white arrowhead), parallel vessel pairs (circle) and expected branches from the internal (asterisk) and lateral thoracic arteries (hollow arrowhead) can be observed in the images. The yellow dashed lines in the ML MIPs show the z-coordinate at which the AP slices of the SOS maps are presented in **d.** The dashed yellow circles in the SOS maps correspond to the contour of the bowl surface. **e**, ML MIPs of the deeper part of the breast for reconstructions using the 1-SOS, 2-SOS or full-SOS model reconstructions. The breast cup contour which corresponds to the surface of the breast is indicated by the white dashed lines. Solid arrowheads highlight vascular structures that are improved with the full-SOS compensated reconstruction. All PA images in this figure are amplitude modulated.

Fig. 4a shows depth color-coded anterior-posterior (AP), medio-lateral (ML) and cranial-caudal (CC) MIPs of the full-SOS compensated PA reconstruction at 800 nm of the right breast of volunteer 1. The images reveal complex vasculature throughout the well-centered breast with a high contrast and a wide and uniform field-of view. (Supplementary figure A7 shows the effect of post-processing using Frangi filtering and AIM on images, which is to suppress background noise and enhance visualization of vessel-like structures.) The superficial vessels (0–0.35 cm deep) located within the dashed box in the AP MIP, are shown in the zoomed in view in Fig. 4b. The intensity profiles of three vessel cross-sections, fitted with a Gaussian, show that vessels with diameters of at least 0.72 mm and larger are depicted.

The full-SOS compensated 3D PA images for the three other volunteers are shown in Fig. 4c, with wavelengths indicated in the images. For all cases, the nipple is visible at the surface (white arrow) and large vessels, presumably branches of the internal (asterisk) and lateral thoracic arteries (hollow arrowhead), show branching formations a few mm beneath the skin. Several deep vessels are also visible; the deepest vessels can be traced up to 34 mm, 48 mm, 43 mm and 42 mm depths for the four volunteers respectively. Several deep vessels are oriented perpendicular to the chest wall (see ML view of volunteer 3) and may be intercostal arteries that feed the deep breast parenchyma or they may originate from superficial veins that drain to the center of the breast [47]. Another observation is that two vessels run closely in parallel or revolve around each other at several locations (circle). These likely are the venous and arterial anatomies that parallel each other [48], especially for intercostal, axillary and internal thoracic vascular pathways.

Fig. 4d shows slices of the FWI-reconstructed SOS images acquired from the UST measurements on volunteers 2, 3, and 4. Large-scale to mid-scale acoustic property heterogeneity is seen with sound speed variations in the range from 1415 to 1560 m/s, which match with values expected in the human breast [49], [50]. The three volunteers show different SOS distributions within the breast, which could indicate different breast parenchymal patterns [51], [52]. The homogeneous and relatively low sound speed in volunteer 2 indicates a fatty breast. The inhomogeneous SOS distribution in volunteer 3 is likely a breast where fibrous or glandular tissue is interwoven with fat. The SOS distribution in volunteer 4 points to a centrally located region of dense fibroglandular tissue surrounded by a layer of fat tissue.

The effect of using our sophisticated SOS model in the PA inversion was investigated by comparing PA reconstructions from the full SOS image from the UST measurements (full-SOS) with a single SOS value (1-SOS) and two different SOS values outside and inside the breast (2-SOS). Fig. 4e shows ML MIPs of volunteers 2–4. To examine deeper-lying vasculature, the first 25 mm were digitally peeled off in the reconstructed volumes (Fig. 4e). These MIPs show that the 1-SOS model only results in groupings of higher intensity pixels, which appear to merge in the 2-SOS approach to form elongated structures rising above noise. In contrast, the full-SOS compensation results in a marked improvement in image sharpness, increased vessel continuity (indicated with solid arrowheads), and the emergence of additional structures, some located at greater depths in the breast. The green semi-circle in the deep breast of volunteer 3 is an artifact that only appears in measurements with cup sizes seven and eight. It originates from the PA signals generated on the surface of the imaging bowl that are back-reflected from the breast surface and detected.

### 4.5 Multi-wavelength in vivo imaging

**Fig. 5:**
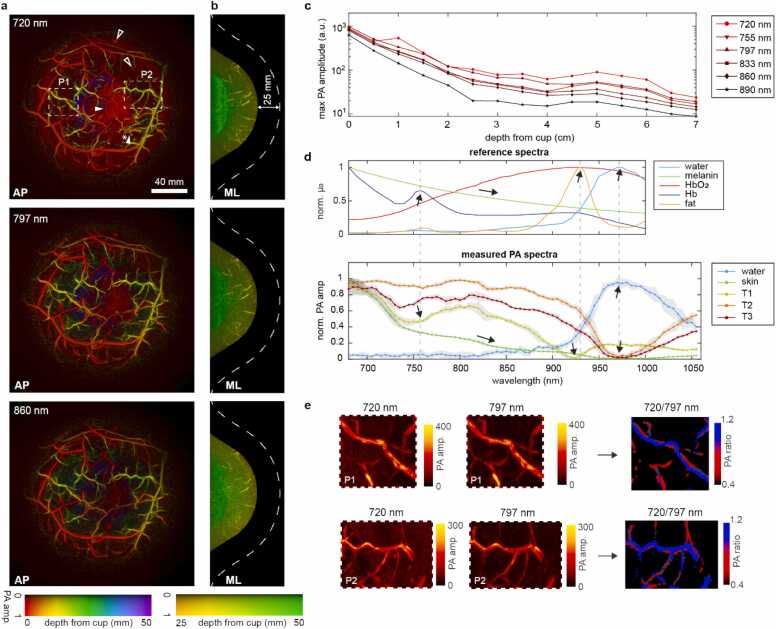
Multispectral in vivo imaging**. a,** Amplitude intensity modulated (AIM) AP PA MIPs of the left breast of a healthy breast (volunteer 3) for three out of the six wavelengths measured with. All images are normalized to their own maximum value. The solid arrowhead in the first image points to the nipple, the hollow arrowheads to birthmarks and the asterisk to a presumably vascular structure. All these indicated structures strongly decay in intensity with increasing wavelength. **b**, ML MIPs of the same PA reconstructions, but only revealing the structures at a depth of 25 mm and beyond. the maximum PA intensity as a function of depth from the cup surface for all six wavelengths. **c**, The maximum PA intensity encountered in 5 mm thick layers going radially inwards from the breast surface for the six different wavelengths imaged with. **d**, PA spectra corresponding to the five peaks in the time trace recorded by the lowest detector in the bowl during a measurement with the bowl static and the laser sweeping over the full wavelength range. **e** AP and CC MIPs of two ROIs containing parallel vessel pairs (P1 and P2). The locations of the pairs in the reconstructed volume are outlined in a. The 720/797 nm ratio images of the segmented vessel pairs show that one vessel contains more oxygen than the other. (AIM was not use on data for developing **c, d** en **e**.).

The left breast of volunteer 3 was imaged with a multi-wavelength measurement sequence (720, 755, 797, 833 and 860 nm) in a measurement time of 6 min. AP MIPs of the PA reconstructions with 720, 797 and 860 nm excitation (Fig. 5a) reveal the familiar vascular architecture, but also melanin-rich structures at the nipple (white arrowhead), the skin and birthmarks on the skin (hollow arrowhead). The PA intensity of these latter structures decays with increasing wavelengths as expected from the known monotonically decreasing melanin absorption spectrum [53]. An interesting observation pertains to a structure located a few millimeters beneath the skin (white arrowhead with asterix). This structure is clearly visible in the 720 nm measurement but becomes indistinguishable at 860 nm. The distinctive form suggests that it is a blood vessel, and its diminishing visibility at longer wavelengths implies that it carries blood with a relatively lower oxygenation level. Fig. 5b reveals the vascular structures at depths of 25 mm and beyond as a ML MIP for the same three wavelengths as in Fig. 5a. The signal-to-noise ratio (SNR) of the vessels decreases with increasing wavelength. The radiant exposure is not same for all wavelengths but follows Fig. 2a. While a vessel in the 720 nm measurement (9 mJ cm^−2^) was traced down to a 48 mm depth, the same vessel was traced down to 43 mm in the 797 nm measurement (7.5 mJ cm^−2^) and down to 39 mm in the 860 nm measurement (6.7 mJ cm^−2^). The decaying SNR with wavelength is also seen in Fig. 5c, which plots maximum PA intensity as a function of depth encountered in 5 mm thick layers going radially inwards from the breast surface. This behavior can be attributed to the wavelength-dependent laser output energy with progressively lower energies at longer wavelengths, combined with the simultaneous increase in (coupling) water absorption at the longer wavelengths until its eventual domination in the near-infrared wavelength regime. The increase in intensity after 40 mm depth can be attributed to the reflection artefact described earlier (this volunteer has cup size 8; cf. Fig. 5b), together with an enhanced sensitivity due to focusing effect of the hemispherical detection aperture towards the center of the bowl. The influence of noise and interference signals begins to dominate at a larger depth around 60 mm.

Spectral signatures of tissue chromophores can also be observed in the recorded PA signals. The amplitudes of five peaks in the time trace recorded by the transducer at the lowest point in the bowl during a wavelength sweep measurement on the left breast of volunteer 3 are plotted as a function of wavelength in Fig. 5d. The five peaks were selected as follows: one was located at a time coordinate corresponding to the water in the imaging bowl, one at a coordinate corresponding to the skin, and the remaining peaks (T1, T2, T3) at later time coordinates belonging to tissues lying deeper in the breast. The time trace and the selected peaks can be found in the Supplementary Figure A5. The behavior of the five peaks individually as function of wavelength demonstrates the influence of the spectra [53] of the prominent chromophores water, melanin, hemoglobin and fat (see Fig. 5d). While the absorption spectrum of water is directly recovered from the peak corresponding to water, the peaks at coordinates inside tissue show the integrated effect of the spectra of various chromophores encountered in the breast on the spherical surface centered on the element and at distance (*v* × *t*). The effect seen is that of spectral coloring by various tissue chromophores [54], [55], for example in the spectrum of T1, the local minimum at 755 nm corresponds to the local peak of Hb at the same wavelength, while the local minimum around 930 nm corresponds to the peak absorption in fat at the same wavelength.

Two parallel vessel pairs (P1 and P2) present in the breast show in the AP MIP within the ROIs outlined by the white dashed lines (Fig. 5a). Fig. 5e presents the zoomed-in AP and CC views of these ROIs. Ratio imaging was performed with images at 720 and 797 nm of these vessel pairs. For this, the ROI-PA image at 797 nm was thresholded to extract the vessel structures and on the non-thresholded voxels, the ratio between the intensities at 720 nm and 797 nm was computed and visualized as an MIP in Fig. 5e, where blue color indicates ratio values above 1 (intensity at 720 nm is higher than at 797 nm) and red below 1. These ratio images consistently show higher ratio values in the one vessel than in the other from which we can conclude that one is an artery and the other a vein.

## 5 Discussion

We have developed the breast imaging system PAM3, that for the first time combines multi-wavelength PA tomography with US tomography in hemispherical geometry. The system is a major technological improvement over the state-of-the-art due to the following advancements in critical hardware and software sub-systems: 1) the dual laser and optical parametric oscillator (OPO) units have a combined output with the highest intensity pulses in their class of laser, from 450 mJ/pulse to 230 mJ/pulse between 680 nm to 1060 nm, 2) the light delivery approach using optical fiber bundles to provide 40 injection points distributed appropriately and with diverging optics, leads to homogeneous exposure with high fluence on the breast surface, 3) the 512 US transducers demonstrate a 123% fractional frequency reception bandwidth centered at 1 MHz; the large active area and carefully designed matching/backing layers realize an MDP of 0.3 Pa which is the lowest reported in literature for photoacoustic breast tomography, 4) the specially designed US pulser-receiver units provide 512-channel low-noise fully programmable electronic I/O modules, 5) fully three-dimensional sound-speed imaging is achieved using FWI of UST measurements, enabling accurate and spatially resolved reconstruction of sound-speed distributions in the heterogeneous breast; and 6) the novel PA image reconstruction method using an accelerated, iterative, first-order optimization procedure [35] can utilize the reconstructed SOS image in PA reconstruction, as well as take into account the spatial and temporal detector responses.

The system was tested and optimized on a specially developed suite of task-based test-objects [37] and a sophisticated PA-US breast phantom [39], [46], and its capabilities and functionalities demonstrated on healthy human volunteers. The 3D SOS images from the UST mode on human breasts revealed large-scale and medium-scale inhomogeneities with values and 3D distributions as can be expected in the breast. The SOS image was used to correct perturbations to the wavefront in PA image reconstruction. The combination of this approach with the technical improvements mentioned above has enabled *in vivo* results with imaging depths up to 48 mm, wide field of view and excellent contrast in the visualization of vascular morphology in the breast at multiple wavelengths. The imaging depth is based on continuous traceability of vessel-like structures in the reconstructed images. PA signals are detectable beyond this depth (Fig. 5c), but these cannot be reliably attributed to vascular structures. While the deepest imaging was recorded at 720 nm at the highest radiant exposure on the breast surface from our laser, this is not necessarily the optimal wavelength for photoacoustic imaging. Imaging depth depends on skin pigmentation, skin characteristics and breast composition in complex ways. To ascertain an optimal wavelength for best penetration is challenging since a large number of volunteers will be required to take the biological variability of the above characteristics into account. An additional confounding factor, is breast physiological status, since hormonal shifts at different phases of the menstrual cycle affect vascularization, water content and tissue density affecting both optical properties and acoustic properties of the organ in largely unknown ways [56].

We also demonstrated that the PAM3 system can perform multi-wavelength PAT *in vivo* studies. It requires 5 min for the standard imaging protocol, which includes 5 wavelengths measured with 4 averages, acquired at 10 Hz. Future work will be in developing and advancing quantitative PAT (QPAT) image reconstruction algorithms for extracting measures of blood oxygen saturation in tissue. The ability to estimate this parameter from tumor vasculature is thought to be key to imaging cancer reliably [57]. Further, multi-wavelength measurements can also allow the estimation of concentrations of other tissue chromophores in the breast such as lipid and water, which are thought to be different between tumor and healthy tissues [21]. A key enabling step towards quantitative imaging has been achieved to solve the acoustic part of the inverse problem accurately with the use of the newly developed FWI-based SOS image from the UST mode. This allows for the imaging of the heterogeneous distribution of speed of sound in the breast which allows the SOS compensation to be performed.

In addition to improving PAT reconstructions, the high-resolution, quantitatively accurate 3D SOS images also provide important anatomical information on the distribution of adipose and fibroglandular tissue that complements the anatomical information on the vascular tissues provided by the PAT images. Furthermore, SOS contrast has been shown to hold great diagnostic potential for tumor identification and characterization and breast density characterization, in particular when combined with acoustic attenuation contrast [58], [59], [60], [61], to which we plan to extend our FWI schemes. Another future research will be to accelerate them to match the spatial resolution of PAT (cf. Figure A5 in the Supplementary Material), and the development of algorithms to image US reflectivity with PAM3 data, because echogenicity contrast is one of the mainstays of standard US imaging and is showing much promise in UST [58], [59], [60], [61]^.^ Reducing the current computing time of 2.5 h for the SOS-compensated PAT image reconstruction significantly will be another topic for future methodical research, for which we will investigate the use of redatuming [62], similar multi-grid schemes as in FWI, and machine-learning [63], [64].

While the potential of PA and US tomography in the context of breast cancer has been shown [4], [6], [7], [8], [9], [10], [11], [12], [13], [14], [15], [16], [17], [18], [19], [20], [21], [22], [23], [24], [25], [26], [27], [28], [29], [30], [31], [32], [33], [34], [35], [36], [37], [38], [39], [40], [41], [42], [43], [44], [45], [46], [47], [48], [49], [50], [51], [52], [53], [54], [55], [56], [57], [58], [65], [66], [67], [68], [69], [70], [71] it is important to acknowledge that there are still gaps in understanding and interpreting PAT and UST breast images. Further, the precise roles for PAT and UST imaging in the breast cancer imaging paradigm, whether in screening, diagnosis, treatment planning, neoadjuvant chemotherapy monitoring or follow-up are not fully known. Whether the method is suitable for specific patient populations such as women with dense breasts is also not known. While recent imaging systems have advanced imaging speed, depth, and clinical feasibility, still many rely on simplified acoustic models or limited integration between acoustic and photoacoustic contrasts. Our PAM3 platform enables fully co-registered, truly tomographic UST and multi-wavelength PAT, together with physics-based reconstruction of heterogeneous acoustic properties. The PAM3 system provides a foundation for conducting focused, hypothesis-driven clinical studies aimed at addressing the lacunae in our understanding and the open questions in our field.

## Author contributions

All authors contributed during the system design phase; FL and MD performed simulations during this phase. EC, MW, FB and TM developed the US detectors, and TK and AM the laser and fiber bundle design. LA developed the DAQ system, RPPM the system mechanics and patient-user interface. MN and LA developed the system control software. TOR contributed to system characterization and extracted reconstruction parameters. FL developed reconstruction codes, under guidance of BC. JB, GB, supervised by JJ, developed codes to pre-process the recorded data before the reconstruction. MD designed the human study protocol with inputs from LFGO, SA, JV and SM. MD wrote the medical device dossier with inputs from LA, RPPM, MN, and did laser safety calculations reviewed by SA. SCK prepared risk analysis and other documents for the ethical review committee under the supervision of SA with inputs and contributions from MN, MD, RPPM. MD, SCK and EB developed test-objects and phantoms and conducted measurements on these. MD and SCK performed the healthy volunteer measurements. FL reconstructed images. MD and SCK analyzed the images with inputs from FL. BdS post-processed the images after reconstruction. MD wrote the paper with SM and FL, with inputs from all co-authors. FL, BC, LFGO and SM reviewed the paper. SM, BC, JJ, WMK, MF, TM, JV and AM acquired financial support, and SM directed the project.

## CRediT authorship contribution statement

**F. Brochin:** Writing – review & editing, Supervision, Resources, Methodology, Investigation, Funding acquisition, Conceptualization. **M. Nanninga:** Writing – review & editing, Software, Methodology, Investigation, Formal analysis, Data curation. **L.F. de Geus-Oei:** Writing – review & editing, Supervision, Funding acquisition, Conceptualization. **R.P. Pompe van Meerdervoort:** Writing – review & editing, Validation, Methodology, Investigation, Formal analysis. **S.H. Aarnink:** Writing – review & editing, Resources, Project administration, Methodology, Investigation, Formal analysis. **L. Alink:** Writing – review & editing, Validation, Software, Methodology, Investigation, Formal analysis, Data curation. **S.C. Kruitwagen:** Writing – review & editing, Validation, Methodology, Formal analysis, Data curation. **F. Lucka:** Writing – original draft, Visualization, Supervision, Software, Methodology, Investigation, Formal analysis, Data curation, Conceptualization. **M. Dantuma:** Writing – original draft, Methodology, Investigation, Formal analysis, Data curation, Conceptualization. **T. Kasponas:** Writing – review & editing, Methodology, Investigation. **S. Manohar:** Writing – original draft, Supervision, Resources, Project administration, Investigation, Funding acquisition, Conceptualization. **M. Wilm:** Writing – review & editing, Supervision, Methodology, Formal analysis, Conceptualization. **B. Cox:** Writing – review & editing, Supervision, Methodology, Investigation, Funding acquisition, Formal analysis, Conceptualization. **E. Coffy:** Writing – review & editing, Validation, Software, Resources. **J. Veltman:** Writing – review & editing, Supervision, Methodology, Investigation, Formal analysis. **G. Bordovsky:** Writing – review & editing, Software, Methodology, Formal analysis. **J. Jaros:** Writing – review & editing, Resources, Investigation, Funding acquisition, Conceptualization. **J. Budisky:** Writing – review & editing, Software, Formal analysis. **W. Muller Kobold:** Supervision, Resources, Project administration. **E. Bosman:** Writing – review & editing, Validation, Methodology. **A. Michailovas:** Writing – review & editing, Supervision, Resources, Investigation, Funding acquisition, Formal analysis, Conceptualization. **B. De Santi:** Writing – review & editing, Visualization, Software, Investigation, Formal analysis. **T. Martinez:** Supervision, Resources, Methodology, Investigation, Formal analysis. **T.J.P.M. Op ’t Root :** Writing – review & editing, Software, Formal analysis.

## Data and Code availability statement

The measured PAT and UST data that underlie this publication will be shared via a University of Twente server together with relevant meta-data, and in line with FAIR (Findability, Accessibility, Interoperability, and Reusability) principles. The reconstructed PA and SOS images of the SOS phantom and the volunteers and Matlab codes to produce the figures derived from them are available on Zenodo [72]. The Matlab codes for reconstructing PA and SOS images will be made available via GitHub in a form that respects intellectual property within the consortium.

## Declaration of Competing Interest

The authors declare that they have no known competing financial interests or personal relationships that could have appeared to influence the work reported in this paper.

## Data Availability

Data will be made available on request.
