# Supplementary material for: Hybrid three-dimensional full-view multi-wavelength photoacoustic and ultrasound breast tomography
Source: Photoacoustics. 2026 Jun 13;50:100847. doi: 10.1016/j.pacs.2026.100847 (PMC13311291; doi:10.1016/j.pacs.2026.100847)
Supplement: Supplementary file 1 — Supplementary material [file mmc1.docx]

**Supplementary material to: Hybrid three-dimensional full-view multi-wavelength photoacoustic and ultrasound breast tomography**

**
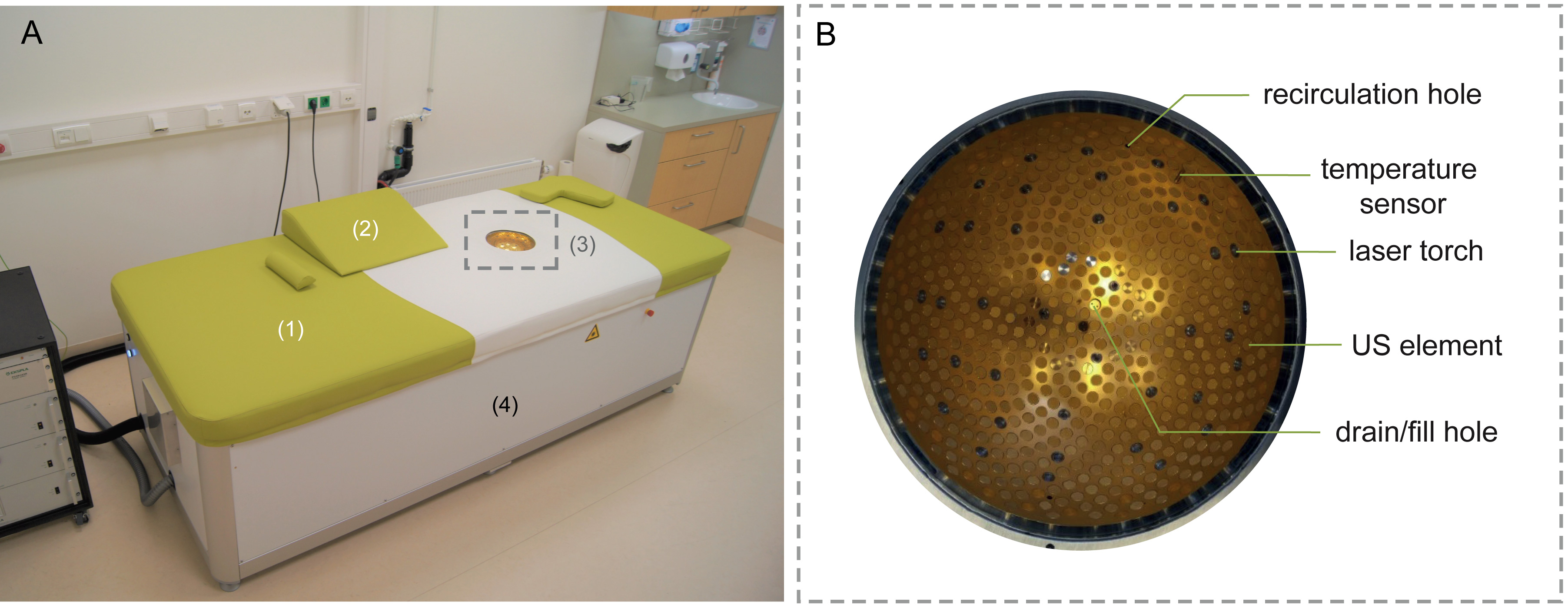
Figure A1.** (A) Photograph of the PAM3 system with the bed (1) on which a woman can lie in prone position. Pillows (2) can be used to position the subject comfortably, and so as to have as much breast tissue available inside the imaging bowl (3). Most of the hardware is hidden behind the panels (4). (B) Photograph of top-view of the imaging bowl, showing the inserts in the spiral pattern.


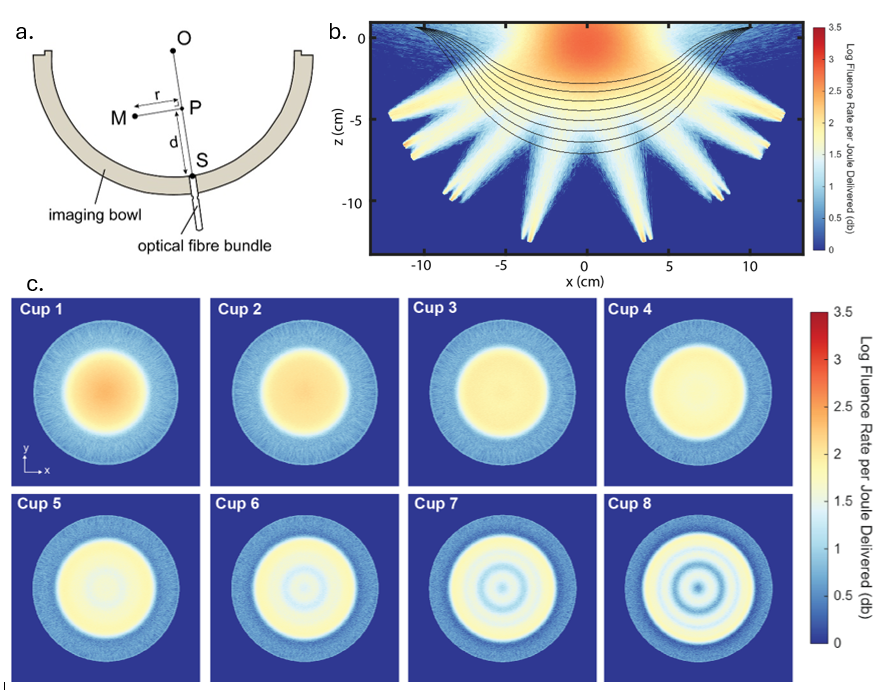


**FigureA2.** (*a*) Illustration showing the source (S), measurement point (M), origin (O) and the projection point (P) in a cross section of the imaging bowl to clarify the model. (*b*) The fluence rate, at a wavelength of 797 nm, in the imaging bowl at y=0 resulting from our model. The dashed lines show contours of the cup sizes. (*c*) The fluence rate on the surface of the cups as seen from the bottom of the imaging bowl. The model incorporates all the 101 bowl rotations resulting in a total of 4040 simulated light sources. Note that the 3D dataset representing the cup’s surface is projected along the z-axis. Pixels located on the upper portions of the cup contribute more significantly to the projection, resulting in stronger values along the edges compared to the center. This visual effect becomes more pronounced with larger cup sizes, which are inherently less flat.


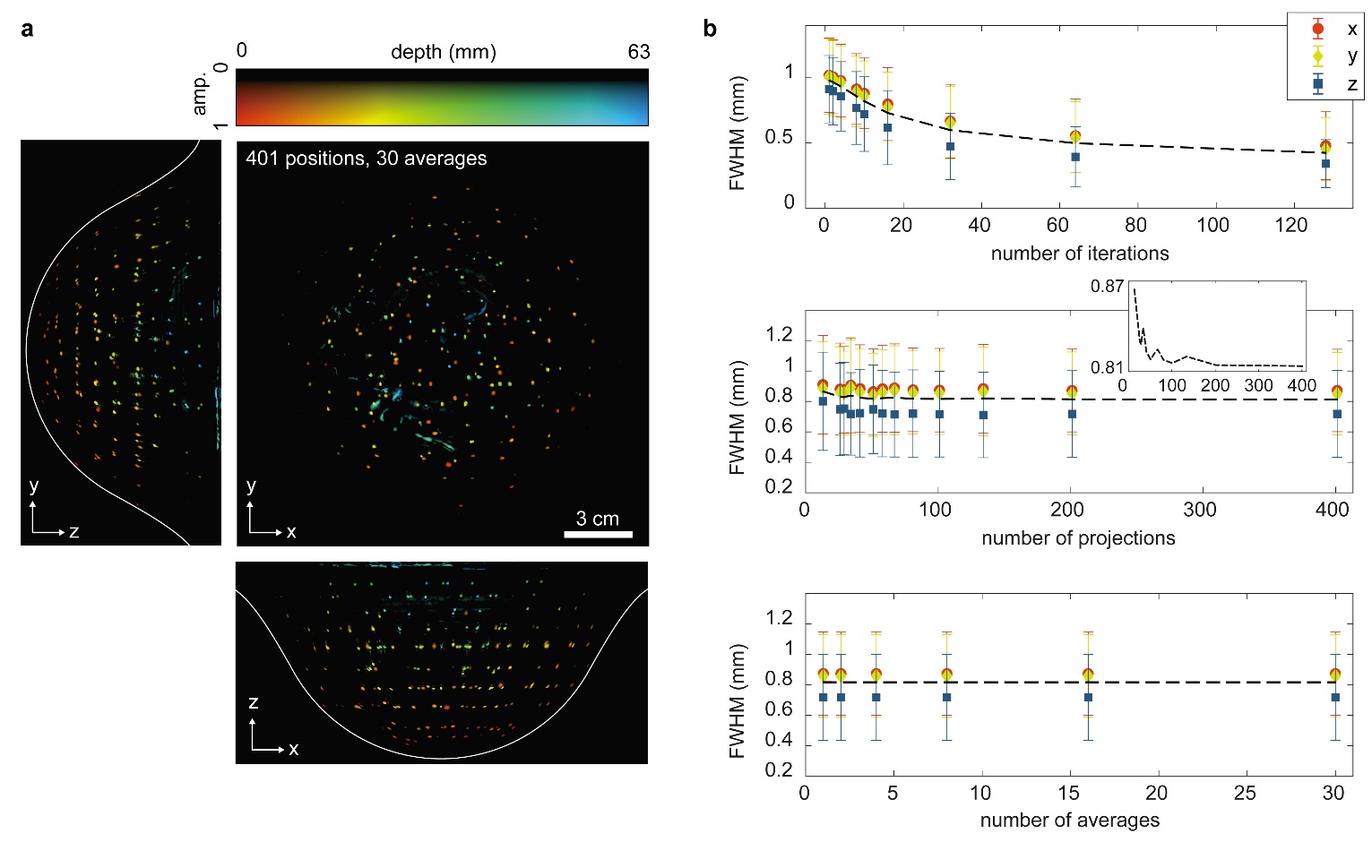


**Figure A3.** Depth color-coded MIPs from an oversampled measurement (800 nm, 401 projections, 30 averages, 10 iterations) on the PSF object. (B) The average FWHM of the Gaussians fitted to 210 reconstructed PSFs plotted for the three reconstruction planes as a function of reconstruction iterations, measurement projections and averages. Error bars indicate standard deviations and the black dashed line is the mean of the FWHM in the three dimensions. The mean curve belonging to the number of projections is shown additionally in an insert to show its decay.


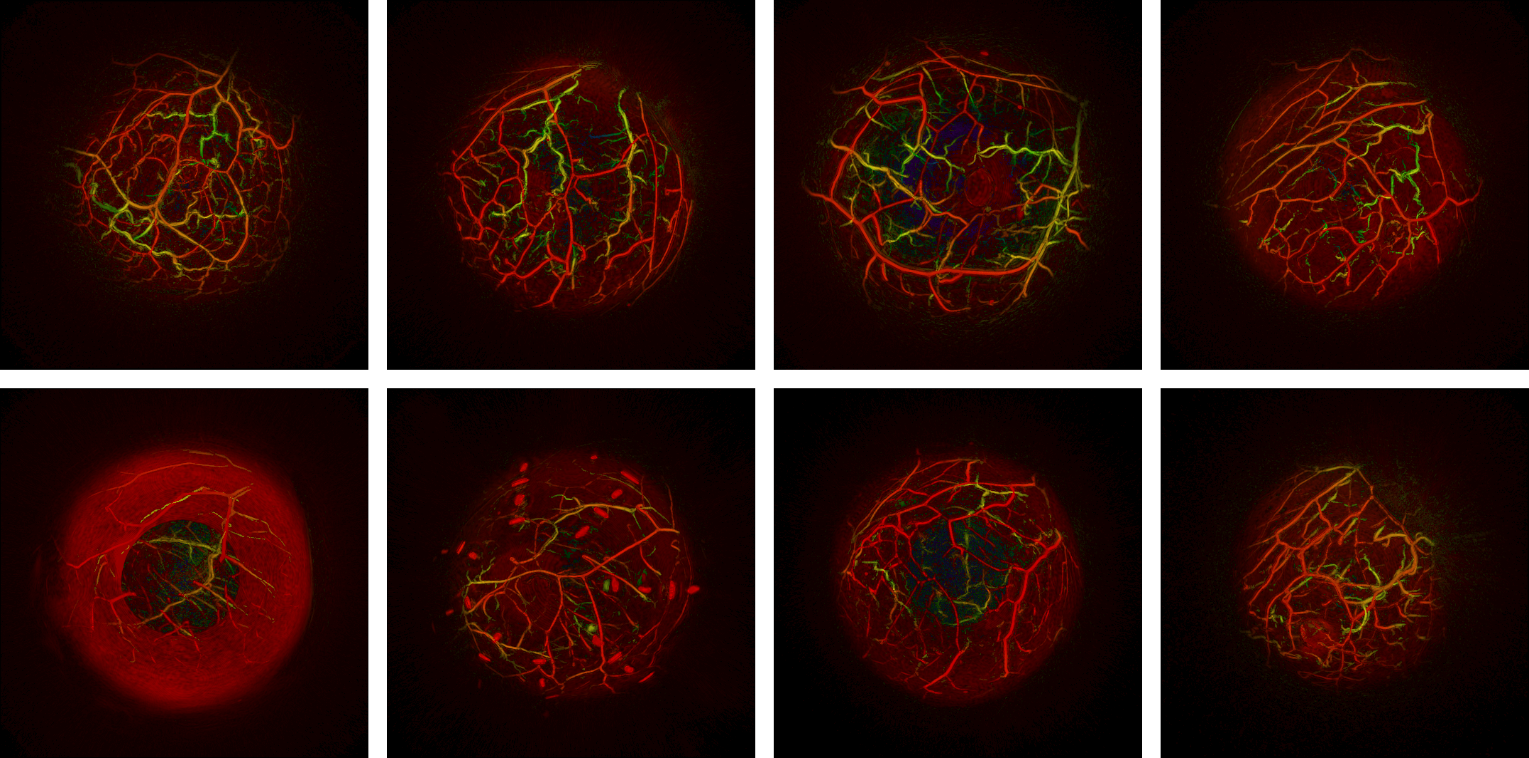


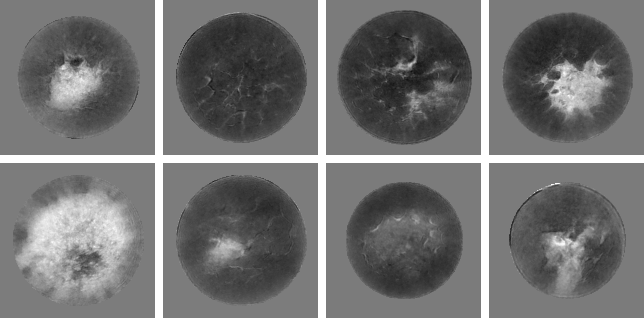
**Figure A4.** Photoacoustic AP MIPs (top), and SOS slices (bottom), of one breast from each volunteer included in the study. The top row shows volunteers 1-4 (left to right) also shown in Figures 4-5 in the main manuscript.


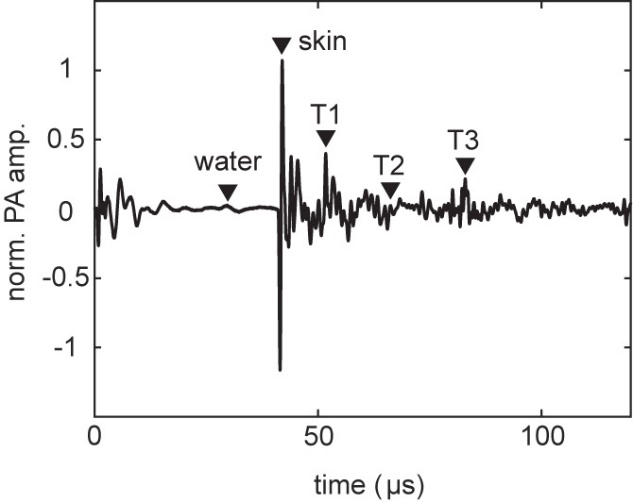


**Figure A5:** PA time trace recorded by the transducer at the bottom at the imaging tank during the measurement on volunteer 016. Peaks that were used to plot the spectra in Figure 5d are highlighted.

**Time-of-flight (TOF) Ultrasound Tomography**

To initialize the FWI on the coarsest grid, an SOS image reconstructed by a time-of-flight (TOF) method is used: For each emitter-receiver transducer pair that is oriented to each other such that a sufficiently strong transmitted wave can be recorded, the first arrival time is extracted from the US time trace [1] and its difference to the corresponding arrival time from a water-only calibration measurement is computed. This arrival-time difference then becomes the data in a constrained, non-linear regression problem where an image of the refractive index with respect to the SOS of the water in the coupling medium is fitted to it. To compute the arrival time difference for a given refractive index image, a bent-ray approach using two-point ray tracing is used [2], which uses the Matlab functions calcRayLinkForward.m and solveQuasiNewton.m and their subfunctions from the r-Wave Toolbox version 1.1, 2023 [3]. The regression objective consists of a smoothed L1-norm data fitting term (“robust regression”) and, as in FWI, a smoothed TV regularization on the refractive index map. Furthermore, bound constraints that correspond to 1400m/s - 1600 m/s are added. To solve the regression problem we use 20 iterations of a Gauss-Newton scheme that solves a series of surrogate problems where the non-linear mapping between refractive index map and arrival time difference is linearized around the current refractive index map (“absolute linearization, see Ref. [2]) and a weighted cubic regularization (“soft trust-region") is added to regularize the linearization. The L-BFGS method [4] is used with 100 iterations to solve each surrogate problem. To ensure a decrease of the original regression objective, the strength of the cubic regularization is adjusted if an increase is detected and the surrogate problem is solved again. Computing an SOS image on a 1 mm spatial grid takes 20 h on a server with an AMD EPYC 7343 16-Core Processor and 1024 GB RAM memory. Further technical, implementation and calibration details will be described in forthcoming publication.

**Additional system information**

The hemispherical bowl is made of polymethylmethacrylate (PMMA) with inner surface coated with a 200 nm thick reflecting gold layer. Water inlet and outlets are present to fill and drain the bowl with degassed, demineralized and UV sterilized water for acoustic coupling, maintained at 25 ⁰C. The imaging bowl together with all its inserts and the I/O modules is connected to a large race wire bearing driven by a stepper motor and a sprocket to allow continuous rotation of the bowl over 360 degrees.

The DAQ consists of 32 pre-amplification channels leading to four 8-channel analog front end (AFE) chips with 14-bit Analog-Digital Converters (ADC). The time traces amplified by 24 dB, are digitized with 50 MHz and 25 MHz sampling frequencies for the PA and US mode respectively, and transmitted to the DAQ-PC. The US Actuation part is responsible for sending the driving signals to the US transducers. It consists of eight 4-channel Digital-Analog Converters (DACs) to send pre-programmed Gaussian-modulated single cycle sinusoidal pulses with a 1 MHz center frequency to the transducers at a 100 Hz repetition rate, where only one transducer emits and all the others receive.

### Laser and light delivery

Both laser heads in the custom-designed high energy tunable laser unit emit pulse-to-pulse wavelength tunable 4.2 ns pulses at 10 Hz repetition rates. In normal operating mode, the pump lasers are triggered with a 90 ns delay between them, to avoid overlap of the two pulses in time in order to stay under the damage threshold of the fiber bundle (see Fig. 1e). In addition to the double pump laser approach, a novel method for compensating depolarization losses is implemented [5] which contributes to achieving high output powers.

The 40 fiber bundles each have a core diameter of 1.67 mm and have a micro lens array beam spreader at the tip providing a beam profile that can be approximated by a Gaussian function. For a pulse repetition frequency of 10 Hz, the maximum permitted exposure (MPE) of 200 J/m^2^ for skin is only exceeded up to 22 mm from the fiber bundle surface. The MPE for the skin at the most restrictive 680 nm wavelength, could possibly only be exceeded for distances < 22 mm from the fiber bundle. The breast-supporting cups restrict the breast from entering this zone.

## System control and data storage

The measurement sequence can be programmed into the control software by the operator. This allows the number of bowl positions, number of PA excitations per position (averages), US shots per position and the number of PA excitation wavelengths to be varied per measurement. In the example measurement sequence (Figure 1e) for each bowl position, PA measurements are performed at five wavelengths. At each wavelength, two combined light pulses are sent into the bowl; a single combined light pulse comprises the outputs of the two pump lasers (L1 and L2) with an 90 ns delay between them. After each combined light pulse is launched, all 512 detection elements are read out and the measured time-traces stored. Thereafter the bowl rotates to a new position, to repeat the measurement sequence. The repetition rate of the laser is 10 Hz, while that of the US emission is 100 Hz. This permits 9 US measurements to be performed in the interval between two PA measurements.

After the operator has selected the sequence, the operator-PC generates a measurement program which is then automatically programmed on the I/O modules and the trigger module. Once the measurement has started, the recorded PA and US time traces are amplified, digitized and stored on the DAQ-PC. The total recorded data size of a measurement depends on the chosen measurement sequence but is usually in the order of 65 GB for a multi-wavelength tomographic PA-US measurement. After the measurement has finished, all the data is downloaded from the DAQ-PC and uploaded to the image reconstruction PCs for generation of the images.

### Spatial resolution calculation

The spatial resolution of the PAM3 system was assessed from the reconstructed images of the over-sampled PA measurement on the PSF test object, consisting out of 401 bowl rotation steps and 30 averages. Reconstructions were made with all the recorded time traces but also with down sampled sets of the recorded data to investigate the effect of the number of bowl positions and averages on the spatial resolution (see Supplementary Figure A2). No post-processing was applied to the reconstructed images. The spatial coordinates of the centers of all the 213 beads in the test object were manually determined based on an initial reconstruction. Then, for each bead, the nearest local intensity maximum was found around which a ROI of 2x2x2 mm^3^ was selected. In the ROI, Gaussians were fitted to the intensity profiles crossing the beads in *x*, *y* and *z* direction. The full width at half maximum (FWHM) of these Gaussians was then calculated as a value for the spatial resolution. Beads that were closer than 3 mm to another bead were discarded, as these may induce inaccuracies in the Gaussian fitting.

**Speed-of-sound test object**: **Fabrication, characterization, additional results**

The test object consists of two tissue-mimicking regions representing adipose and fibroglandular breast tissue. The adipose region was formed using a nylon breast mold (cup size 8) filled with degassed native gel wax (NGW) containing a 6% paraffin additive to mimic adipose tissue. The fibroglandular region was defined by a paraboloid cavity embedded within the adipose volume and filled with degassed 10% gelatin. Four cylindrical rod-shaped channels with diameters of 4, 6, 8, and 10 mm, filled with water, were embedded within the fibroglandular region to assess spatial resolution and sound-speed contrast.

The acoustic properties of the materials used in the sound-speed test object were characterized using transmission measurements based on a modified insertion method [REF]. Measurements were performed under controlled temperature conditions. Unfocused single-element ultrasound transducers (Olympus NDT, USA) with center frequencies of 1 MHz and 2.25 MHz were used in combination with a needle hydrophone (SN3753 NH1000, Precision Acoustics, UK). The transmitting transducer was driven by a pulser–receiver unit (5077PR, Olympus Panametrics NDT, USA), and received signals were recorded using a preamplifier and oscilloscope.

Material samples corresponding to the adipose- and fibroglandular-tissue-mimicking components of the phantom were prepared with identical geometry and composition. Transmission measurements were performed through both the thin and thick dimensions of each sample by rotating the sample within the setup to vary the acoustic path length. Sound speed was calculated from the measured time-of-flight differences using the standard insertion-method formulation, At a temperature of 24.9 ± 0.2 °C, the measured (min / max / mean±std) sound speeds were (1450.2 / 1455.5 / 1452.5 ± 2.3) m/s for NGW and (1521.1 / 1525.2 / 1522.8 ± 1.7) m/s for gelatin. These values were used to create the reference SOS image used in the validation of the FWI SOS reconstruction shown in Figures 3d-3e in the manuscript.

The geometry of the reference SOS image was constructed as follows: The boundary of the outer compartment is clearly visible in the PA reconstruction and the acoustic medium between the US transducers and this boundary is well characterized as water with an average temperature 24.9°C. Therefore, a PA reconstruction with a 1-SOS model will place the boundary at the right location. Then, the known geometric shape of the outer compartment (breast cup with size 8, for which we have an analytical formula), is manually fitted into the image such that the surfaces match. With a similar reasoning, the inner compartment position is found: The boundary of it is clearly visible in the PA reconstruction. With the outer compartment in place, and it’s SOS fixed to 1452.5 m/s (see above), the acoustic medium between US transducers and the inner compartment boundary is well characterized, and a PA reconstruction with a 2-SOS model will place this boundary at the right location. Again, the known geometric shape of the inner compartment (we have an analytical formula), is manually fitted into the image such that the surfaces match. The four rods are not visible in the PA images, but clearly visible in the FWI SOS gradient image computed at a 3-SOS model (water at 1497.4 m/s, outer compartment at 1452.5 m/s, inner compartment at 1522.8 m/s). With a similar reasoning as above, the acoustic model between the US transducers and the most outward surface parts of the rods is well characterized, and the reflections of the four rods should propagate to the correct locations. The gradient was computed on a resolution of 1mm by averaging 4096 source encoded stochastic gradients. Then, we again fit the known geometric shapes of the rods manually into the image. As a result, we have a geometric description of the reference SOS image from which a segmentation can be derived for any given spatial resolution. The different steps of the construction we described are validated in the Matlab script validate_reference_SOS_model.m placed in the code repository (see above).

To illustrate the potential of UST with the PAM3 scanner using FWI, we extended the standard multi-grid scheme used for the in-vivo data by running an additional 64 iterations on 0.8mm and 0.6mm resolution. While this increases the total computation time from 10h to 68h with the computational servers available to us, one can clearly see in Figure A6 that the SOS transitions at tissue boundaries are better resolved, the quantitative accuracy increases and small-scale structures like the rods become more visible.


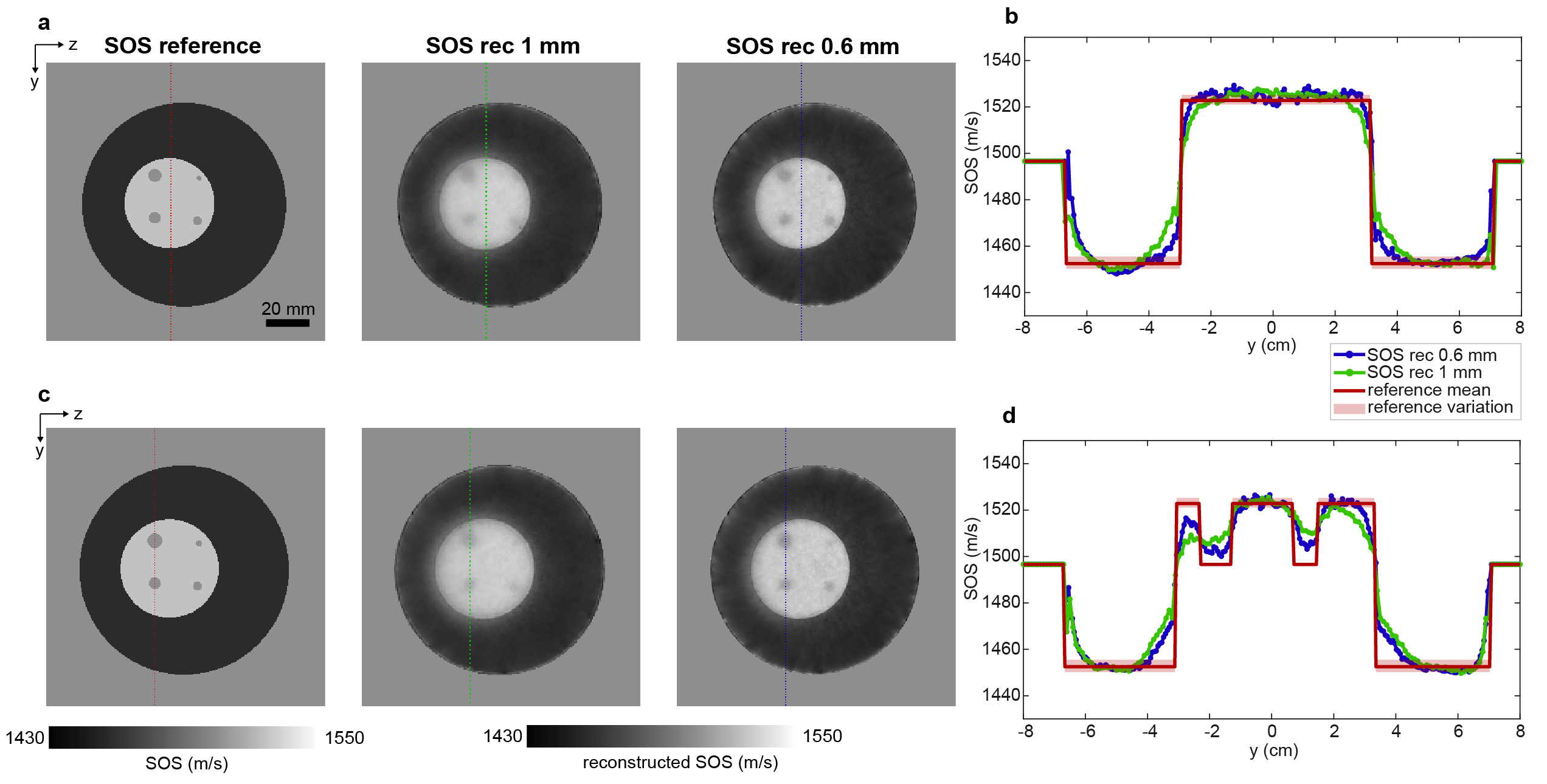
**Figure A6:** Comparison of FWI reconstructions of the SOS test object with a multi-grid scheme that stops at 1mm resolution vs a scheme that stops at 0.6 mm resolution, cf. Fig 3d-3f of the manuscripts. Top and bottom row show different z-slices and line profiles through the same 3D reconstructions. The shaded areas labeled as “reference variation” in the line plots indicate the min-max ranges of the results of the characterization measurements.


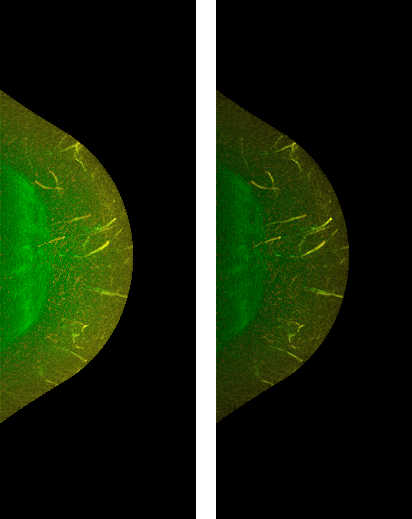

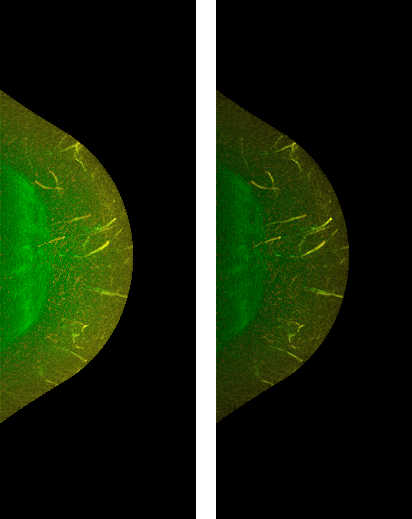


**Figure A7:** Comparison of raw PA image (left) and processed by adaptive intensity modulation using Frangi filtering (right) of volunteer 3, computed with SOS map (cf. Figure 4 e).

**Table A1.** Mean and standard deviations of the fluence rate (at 797 nm wavelength) on breast surface for different cup-sizes.

| **Cup size** | **Mean ± SD (dB)** |
| --- | --- |
| 1 | 1.46 ± 0.94 |
| 2 | 1.43 ± 0.80 |
| 3 | 1.27 ± 0.90 |
| 4 | 1.27 ± 0.77 |
| 5 | 1.30 ± 0.73 |
| 6 | 1.30 ± 0.69 |
| 7 | 1.34 ± 0.69 |
| 8 | 1.24 ± 0.78 |

**Table A2.** Details of image acquisition of all volunteers in the manuscript.

| **Volunteer** | **Wavelengths (nm)** | **Number of views** | **Number of averages** | **Acquisition time (minutes)** |
| --- | --- | --- | --- | --- |
| 1 | 800, 860 | 101 | 12 | 6 |
| 2 | 870 | 101 | 10 | 3 |
| 3 | 720, 755, 797, 833, 860, 890 | 101 | 4 | 6 |
| 4 | 720, 755, 797, 833, 870 | 101 | 4 | 5 |

**Table A3.** Description of Fitzpatrick scale skin types

| Type 1 | Highly sensitive, always burns, never tans. Example: Red hair with freckles. |
| --- | --- |
| Type 2 | Very sun sensitive, burns easily, tans minimally. Example: Fair skinned, fair haired Caucasians. |
| Type 3 | Sun sensitive skin, sometimes burns, slowly tans to light brown. Example: Darker Caucasians. |
| Type 4 | Minimally sun sensitive, burns minimally, always tans to moderate brown. Example: Mediterranean type Caucasians, some Hispanics. |
| Type 5 | Sun insensitive skin, rarely burns, tans well. Example: some Hispanic and some Black individuals. |
| Type 6 | Sun insensitive, never burns, deeply pigmented. Example: Darker Black individuals. |

**Table A4.** Questionnaire used for skin type scaling on the Fitzpatrick scale. Summing the points corresponding to the answers gives the six skin types described in Table A1. I: 0-6 points, II: 6-14 points III: 15-22 points, IV: 22-27 points, V and VI: >27 points


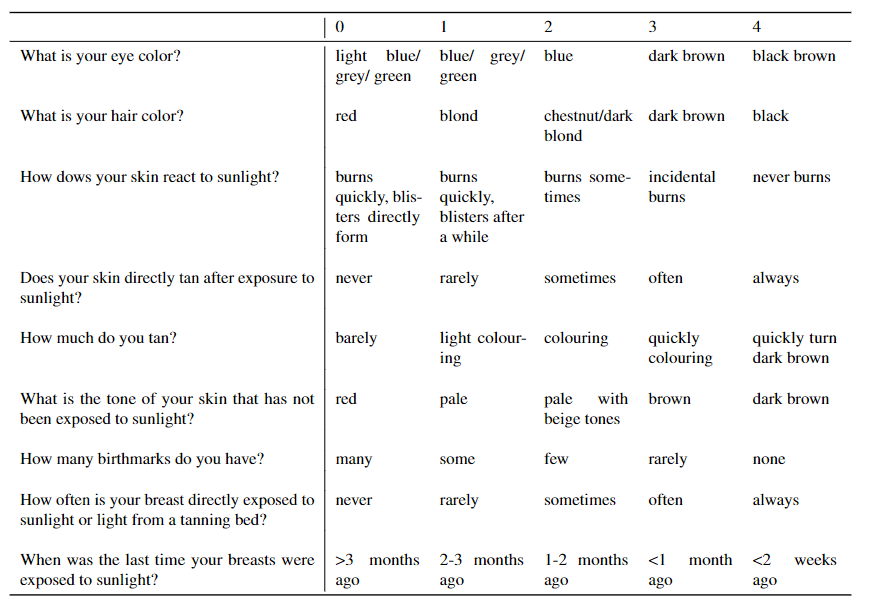


**References**

[1] Li, C., Huang, L., Duric, N., Zhang, H., & Rowe, C. (2009). An improved automatic time-of-flight picker for medical ultrasound tomography. Ultrasonics, 49(1), 61-72.

[2] Javaherian, A., Lucka, F., & Cox, B. T. (2020). Refraction-corrected ray-based inversion for three-dimensional ultrasound tomography of the breast. Inverse Problems, 36(12), 125010.

[3] https://github.com/Ash1362/ray-based-quantitative-ultrasound-tomography

[4] Liu, D. C., & Nocedal, J. (1989). On the limited memory BFGS method for large scale optimization. *Mathematical programming*, *45*(1), 503-528.

[5] A. Michalovas, Depolarization compensator, EU patent EP3712664 (A1), 2020-09-23
